# Advances in the Regulation of Epidermal Cell Development by C2H2 Zinc Finger Proteins in Plants

**DOI:** 10.3389/fpls.2021.754512

**Published:** 2021-09-24

**Authors:** Guoliang Han, Yuxia Li, Ziqi Qiao, Chengfeng Wang, Yang Zhao, Jianrong Guo, Min Chen, Baoshan Wang

**Affiliations:** Shandong Provincial Key Laboratory of Plant Stress Research, College of Life Science, Shandong Normal University, Jinan, China

**Keywords:** C2H2 zinc finger proteins, development, trichome, root hair, salt gland

## Abstract

Plant epidermal cells, such as trichomes, root hairs, salt glands, and stomata, play pivotal roles in the growth, development, and environmental adaptation of terrestrial plants. Cell fate determination, differentiation, and the formation of epidermal structures represent basic developmental processes in multicellular organisms. Increasing evidence indicates that C2H2 zinc finger proteins play important roles in regulating the development of epidermal structures in plants and plant adaptation to unfavorable environments. Here, we systematically summarize the molecular mechanism underlying the roles of C2H2 zinc finger proteins in controlling epidermal cell formation in plants, with an emphasis on trichomes, root hairs, and salt glands and their roles in plant adaptation to environmental stress. In addition, we discuss the possible roles of homologous C2H2 zinc finger proteins in trichome development in non-halophytes and salt gland development in halophytes based on bioinformatic analysis. This review provides a foundation for further study of epidermal cell development and abiotic stress responses in plants.

## Introduction

Cell differentiation and morphogenesis must occur at the correct time and place to ensure the normal growth and development of multicellular organisms (Song et al., 2019; Haas et al., 2020; Su et al., 2021). Plant cell differentiation is precisely and intricately regulated to enable these organisms to successfully complete their lifecycles (Glover, 2000; Tang et al., 2020). Root hairs, stomata, trichomes, and salt glands, the major epidermal structures of terrestrial plants, play essential roles in plant growth, development, and adaptation to environmental stress (Yuan et al., 2019b; Li et al., 2021). The epidermis, a barrier between the plant and the outside environment, is involved in many biological processes, including transpiration, water and nutrient absorption, and resistance to pathogen invasion (Yang and Ye, 2012; Kundu et al., 2018).

Many studies have explored the molecular mechanisms involved in plant epidermal development. This process is well understood for all epidermal components except salt glands. Various types of genes and plant hormones are involved in the development of plant epidermal structures (Shibata and Sugimoto, 2019; Wang et al., 2019c; Nunes et al., 2020). Many transcription factors play important roles in plant epidermal cell development, such as MYB transcription factors (Wang et al., 2019b; Zhang et al., 2019; Xu et al., 2021), bHLH transcription factors (Wang et al., 2019c; Yan et al., 2021), WD40 proteins (Yuan et al., 2019b), HD-ZIP transcription factors (Zhang et al., 2021), and zinc finger proteins (Chang et al., 2018; Yan et al., 2021). C2H2 zinc finger proteins, which play important roles in a variety of biological processes, comprise the largest transcription family in plants and are widely distributed in many species (Sakamoto et al., 2004; Han et al., 2020b; Zhang et al., 2020; Yang et al., 2021). Here, we review recent progress in our understanding of the roles of C2H2 zinc finger proteins in regulating plant epidermal cell development, particularly focusing on several members of this large protein family.

## Materials and Methods

### Phylogenetic Tree Construction and Protein Domain Analysis

For phylogenetic tree construction, the amino acid sequences of C2H2 zinc finger proteins GIS, GIS2, GIS3, ZFP5, ZFP8, and ZFP6 from *Arabidopsis thaliana*, NbGIS from tobacco (*Nicotiana tabacum*), and the Hair protein from tomato (*Solanum lycopersicum*) were obtained from TAIR^1^ and NCBI.^2^ The amino acid sequences of C2H2 zinc finger proteins from *Limonium bicolor* such as LbGIS, LbGIS2, LbGIS3, LbZFP5, LbZFP8, and LbZFP6 were obtained in our laboratory and are shown in Supplementary Table 1, along with the sequences from the other species mentioned above (the genome sequence of *L. bicolor* has been uploaded to NCBI with the accession number PRJNA753199). MEGA 5.1 was used to construct a Neighbor-Joining phylogenetic tree with 1,000 bootstrap replicates. For protein domain analysis, SMART^3^ was used to analyze the motifs in the different protein sequences (Han et al., 2020b).

### C2H2 Zinc Finger Proteins Play Important Roles in Plant Development, Growth, and Stress Resistance

Zinc finger proteins form a finger-like polymorphic spatial configuration by binding Zn^2+^. The many types of zinc finger proteins are named based on the different combinations of Cys and His residues that bind to Zn^2+^ (Zhuang et al., 2020). C2H2 transcription factors are the most abundant and widely studied zinc finger proteins in eukaryotes (Fang et al., 2020). In 1985, the first C2H2 zinc finger protein was identified in *Xenopus* oocytes (Miller et al., 1985), while the first such protein identified in plants was EPF1 in *Petunia*, which was characterized in 1992 (Takatsuji et al., 1992). Since then, C2H2 zinc finger proteins in plants have been extensively studied (Lyu and Cao, 2018; Arrey-Salas et al., 2021).

There are two major differences between C2H2 zinc finger proteins in plants and other eukaryotes. First, plant zinc finger proteins contain long spacers between the adjacent zinc finger structures, whereas in yeast and animals, the zinc fingers are generally clustered together and separated by short spacers (six to eight amino acids) called HC links (Schuh et al., 1986; Han et al., 2020a). Second, most plant zinc finger proteins contain an invariant QALGGH sequence in their zinc finger domains, which is not present in zinc finger proteins in animals or yeast (Schuh et al., 1986; Han et al., 2020a). C2H2 zinc finger proteins usually act as transcription factors that bind to DNA to activate or repress the expression of downstream genes (Takatsuji et al., 1994; Sakamoto et al., 2004; Han et al., 2020a). C2H2 zinc finger proteins in eukaryotes contain one of the most common DNA-binding motifs, a motif of approximately 30 amino acids described as X-X-C-X_(1-5)_-CX_(1-2)_-HX_(3-6)_-H (X represents any amino acid); this motif forms a tight finger-like structure comprising two β-strands and one α-helix (Punta et al., 2011). In addition to DNA, C2H2 zinc finger proteins also interact with RNA and other proteins in plants (Yuan et al., 2018).

C2H2 zinc finger proteins are involved in the growth and development of many plant organs and structures, including flowers (Lyu and Cao, 2018; Zhuang et al., 2020), seeds (Joseph et al., 2014), cell walls (Lyu et al., 2019), trichomes (Chang et al., 2018), cotton (*Gossypium* sp.) fibers (Salih et al., 2019), and root hairs (Ciftci-Yilmaz et al., 2007; Huang et al., 2020; Ming et al., 2020). They are also involved in various biotic and abiotic stress responses (Wang et al., 2009; Zhao et al., 2020), such as resistance to high salinity (Sun et al., 2019), low temperatures (Song et al., 2012; Yu et al., 2014), drought (Cheuk et al., 2020), osmotic stress (Zhang et al., 2016), and reactive oxygen species (Zhang et al., 2014). C2H2 zinc finger proteins improve plant stress resistance by maintaining the proper ionic balance, increasing the levels of osmotic adjustment substances, enhancing antioxidant capacity, and regulating the expression of downstream stress resistance genes *via* the abscisic acid (ABA) or mitogen-activated protein kinase (MAPK) signaling pathways (Wang et al., 2019a; Han et al., 2020a).

### Regulation of Trichome Development by C2H2 Zinc Finger Proteins

Trichomes are specialized epidermal structures with a wide range of morphological types, including branched or unbranched, comprising one or multiple cells (Hauser, 2014; Chang et al., 2019). Unicellular trichomes exhibit a simple structure, commonly lack glands, and are usually unable to secrete secondary metabolites; such trichomes are present in plants such as Arabidopsis, cotton, and cruciferous vegetables (Giordano et al., 2020). By contrast, multicellular trichomes have a complex structure. Some multicellular trichomes are able to secrete various substances, such as I, IV, VI, and VII type trichomes of tomato and the trichomes of *Artemisia annua* and tobacco (Karabourniotis et al., 2020; Schuurink and Tissier, 2020), whereas some lack secretory ability, such as II, III, V, and VII type trichomes of tomato (Yuan et al., 2021). Some trichomes contain glands that secrete secondary metabolites, such as nicotine or toxic substances, to drive away herbivores (Werker, 2000; Schmidt et al., 2011; Konarska and Łotocka, 2020; Pan et al., 2021). The main function of trichomes is to act as a natural defense system to protect the plant from both pests and physical damage (Tian et al., 2012; Sato et al., 2019; Andama et al., 2020). They can also reduce the temperature of the leaf and decrease water loss to enhance plant resistance to abiotic stress (Rao et al., 2015; Karabourniotis et al., 2020). Some trichomes have high economic value. For example, cotton fibers are trichomes located on the ovules of plants that are highly valuable to the textile industry (Tian and Zhang, 2021). Artemisinin, the main ingredient in a medicine used to treat malaria, is synthesized in the glandular trichomes of *A. annua* (Judd et al., 2019; Zhou et al., 2020). In addition, the secondary metabolites produced by the trichomes of certain plant species can be extracted and refined for a wide variety of uses, such as medicinal materials, toiletries, and cosmetics (Zhou et al., 2011; Cao et al., 2020).

The structure of the Arabidopsis trichome is relatively simple; most of these single-celled non-glandular trichomes have three branches (Scutenaire et al., 2018; Wei et al., 2018). The development of Arabidopsis trichomes is closely related to cell fate and nuclear DNA replication (Schnittger et al., 2002). Trichome cells gradually expand after four rounds of replication of genetic material in the nucleus. The trichome cells then elongate vertically to the outside of the leaf surface to form a rod-like structure, and the top of the cell develops to form a conjugate focus that gives rise to three branches. Until the end of differentiation, the top of each branch gradually becomes pointed and assumes a papillary shape (Doroshkov et al., 2019).

The developmental process of the single-celled Arabidopsis trichome is relatively simple, making it easy to observe and perform genetic analysis of this structure. Therefore, Arabidopsis trichome development has become an ideal model for studying plant cell fate (Hung et al., 2020). Arabidopsis trichome development involves four steps: fate determination, initiation, branching, and elongation (Hülskamp, 2004; Schellmann and Hulskamp, 2004; Wang et al., 2019c). The molecular mechanisms involved in this process include a regulatory network composed of numerous transcription factors and other proteins, such as MYBs, bHLHs, WD40 repeat proteins, HD-ZIP, and C2H2 zinc finger proteins (Hülskamp, 2004; Pan et al., 2015; Doroshkov et al., 2019). Similar types of proteins also regulate the development of trichomes in plants such as cotton, tobacco, tomato, and *A. annua* (Chalvin et al., 2020; Wang et al., 2021). Among these, C2H2 zinc finger proteins play important roles in various stages of trichome development in different plants.

### Roles of C2H2 Zinc Finger Proteins in Regulating Unicellular Trichome Development in Arabidopsis

GIS was the first C2H2 zinc finger protein shown to be involved in epidermal cell development in Arabidopsis (Gan et al., 2006), in addition to regulating shoot maturation (Gan et al., 2006). *GIS* is highly expressed in the Arabidopsis stem epidermis and floral meristem, where it plays a key role in controlling the occurrence and development of trichomes. The overexpression of *GIS* led to a higher density of trichomes in inflorescence organs, including the formation of ectopic trichomes on carpels, petals, and even stamens during flower development, while *gis* mutants show significantly reduced numbers of inflorescence trichomes (Gan et al., 2007b). *GIS* is upregulated by gibberellins (GAs) and acts upstream of a key gene for trichome development, *GL1*, *via* the GA signaling pathway (Gan et al., 2006; Liu et al., 2017). In addition, GIS functions downstream of SPY, a repressor of the GA signaling pathway. GIS is also involved in the GA-mediated induction of *GL1* expression in Arabidopsis inflorescences and plays an antagonistic role to the DELLA repressor GA INSENSITIVE (GAI; Gan et al., 2006). In addition, GIS functions downstream of STICHEL (STI) and SIAMESE (SIM), key regulators of trichome branching, and is also regulated by the GA signaling pathway during this process (Sun et al., 2013).

Two GIS homologs, GIS2 and ZINC FINGER PROTEIN8 (ZFP8), also participate in Arabidopsis trichome development (Zhou et al., 2011). The *zfp8* loss-of-function mutant has significantly reduced trichome density on its upper stems, leaves, and branches, and the *gis2* mutant has significantly reduced numbers of trichomes on its flowers. RT-qPCR analysis indicated that the expression of *GIS2* and *ZFP8* is induced by GA (Gan et al., 2007a). Therefore, GIS2 and ZFP8 are required for the regulation of GA-mediated trichome initiation throughout inflorescence development, using a similar regulatory mechanism to that of GIS by the DELLA repressors RGA-LIKE1 (RGL1), RGL2, RGA (repressor of the *ga1-3* mutant), and GAI (Gan et al., 2007b). However, unlike *GIS*, the expression of *GIS2* and *ZFP8* is also induced by cytokinin, and both GIS2 and ZFP8 are involved in the cytokinin-mediated initiation of trichome development in inflorescence organs. GIS2 also functions downstream of SPY and upstream of GL1, thus playing an important role in the cytokinin signaling pathway during trichome development (Gan et al., 2007a).

GIS3 is another GIS-family C2H2 zinc finger protein involved in regulating trichome development in Arabidopsis (Sun et al., 2015). Compared with wild-type plants, the loss-of-function *gis3* mutant produces significantly fewer trichomes on its cauline leaves, lateral branches, sepals, and main stems, while the overexpression of *GIS3* resulted in increased trichome density in sepals, stem leaves, lateral branches, and main inflorescence stems, as well as the ectopic formation of trichomes on carpels. These findings indicate that GIS3 positively regulates the initial development of trichomes on inflorescence stems and floral organs in Arabidopsis (Sun et al., 2015). GIS3 acts downstream of the GA and cytokinin signaling pathways and upstream of *GIS*, *GIS2*, *ZFP8*, *GL1*, and *GL3*. Chemically induced gene expression and chromatin immunoprecipitation analyses further revealed that GIS3 directly targets the promoters of *GIS* and *GIS2* to control trichome development (Sun et al., 2015).

ZFP5 positively regulates trichome development in Arabidopsis *via* the GA signaling pathway. Compared with the wild type, the density of trichomes on the secondary branches, cauline leaves, calyx, and main inflorescence of the *zfp5* mutant is significantly reduced, while the density of trichomes on the secondary branches and inflorescences of *ZFP5*-overexpressing plants significantly increased. Like *GIS*-overexpressing plants, ectopic trichomes were also found on the carpels, petals, and other inflorescence organs of *ZFP5*-overexpressing plants (Zhou et al., 2011). Molecular and biochemical analyses demonstrated that ZFP5 regulates trichome development upstream of GIS, GIS2, ZFP8, GL1, and GL3 and that *ZFP8* is the direct target of ZFP5. Further analysis showed that ZFP5 plays an equivalent role to GIS and GIS2 in regulating trichome formation, which is consistent with the finding that of all of the ZFPs, ZFP5 is most similar to ZFP8 and GIS, as revealed by phylogenetic analysis (Zhou et al., 2012).

Phylogenetic analysis of ZFP protein sequences showed that ZFP6 and ZFP5 are closely related (Zhou et al., 2013). In addition, *ZFP6* has a similar expression pattern to *ZFP5*, with the greatest expression in roots, mature stems, and lateral branches. The number of trichomes on the sepals, secondary branches, and main inflorescence axis of the *zfp6* mutant is significantly reduced. On the contrary, the number of trichomes in the leaves of secondary stems, secondary lateral branches, and the main inflorescence axis was significantly higher in *ZFP6*-overexpressing lines compared to the wild type. Ectopic trichomes were also found on the carpels and petals of *ZFP6*-overexpressing plants. ZFP6 was also shown to regulate trichome differentiation through the GA and cytokinin signaling pathways. Further molecular and biochemical analyses showed that ZFP6 acts upstream of ZFP5 and GIS.

ZFP1 is the most recently reported C2H2 zinc finger protein involved in Arabidopsis trichome development (Zhang et al., 2020). Compared to wild-type plants, the number of trichomes on the stem leaves, secondary lateral branches, and main stems of *zfp1* loss-of-function mutants is significantly reduced, while the overexpression lines had the opposite phenotypes. Further analysis demonstrated that ZFP1 mediates trichome development *via* cytokinin signaling and that ZFP1 plays a role upstream of the key trichome initiation factor GL3.

### Roles of C2H2 Zinc Finger Proteins in Regulating Multicellular Trichome Development in Tobacco and Tomato

The ectopic expression of *GIS* also affects trichome development in tobacco *via* its effects on the GA signaling pathway; however, the role of GIS in tobacco differs from that in Arabidopsis. Arabidopsis GIS stimulates trichome development on inflorescences, stems, and floral organs, while in tobacco, it also regulates trichome development on leaves (Liu et al., 2017). NbGIS positively regulates the development of glandular trichomes in tobacco (Liu et al., 2018). Compared with the wild type, plants overexpressing *NbGIS* showed a higher density of glandular trichomes on leaves, main stems, lateral branches, and sepals, whereas NbGIS:RNAi plants had the opposite phenotypes. NbGIS responds to GA signals and plays a negative role in regulating GA biosynthesis, which significantly affects the accumulation of GA in tobacco. NbGIS regulates the initiation of glandular trichomes in tobacco by regulating the expression of the downstream *NbMYB123*-like gene (Liu et al., 2018).

Homologs of *ZFP8* have also been reported in other species. The transgenic expression of *JcZFP8* (cloned from *Jatropha curcas*) in tobacco resulted in longer and denser trichomes on the leaves and flowers compared to wild-type tobacco (Shi et al., 2018b). To confirm that the phenotype of trichomes in transgenic tobacco was due to the overexpression of *JcZFP8*, the *JcZFP8* gene in the transgenic line was knocked out using CRISPR-Cas9. The trichomes of the knockout line were similar to those of the wild type, confirming that the transgenic expression of *JcZFP8* caused major changes in the structural characteristics of tobacco trichomes (Shi et al., 2018b). RNA-seq of the transgenic lines and the wild type showed that JcZFP8 upregulated the expression of auxin signal transduction-related genes (such as *AUX1* and *ARF*) and downregulated the expression of genes related to the signal transduction of GA, ABA, jasmonic acid (JA), and salicylic acid (SA). In transgenic *JcZFP8*-expressing tobacco, the auxin signaling pathway predominantly promoted the initiation of trichomes. SA negatively regulates trichome development (Traw and Bergelson, 2003), suggesting that JcZFP8 also regulates trichome development by downregulating the expression of SA signaling pathway-related genes (Shi et al., 2018a). In addition, the *JcZFP8*-expressing plants showed a dwarf phenotype similar to that of GA mutants, which in both cases could be alleviated by the application of exogenous GA. Thus, the site of action of JcZFP8 is thought to be related to the GA signaling pathway (Shi et al., 2018b). Transcriptome analysis showed that *SlCycB2*, which is essential for the formation of trichomes in transgenic tobacco, was significantly upregulated in these plants. Many *MYB* genes related to trichome development were also upregulated, indicating that JcZFP8 regulates trichome development by inducing the expression of *MYB* and *CycB*-related genes (Shi et al., 2018b).

The *Hair* gene encodes a zinc finger transcription factor with a single C2H2 domain in tomato (Chang et al., 2018). *Hair* is involved in the formation of type I trichomes in tomato. Yeast two-hybrid and pull-down assays showed that Hair interacts with Woolly (Wo) and SlCycB2, both of which positively regulate the development of multicellular epidermal hairs in tomato. These three proteins may therefore form a Hair–Wo–SlCycB2 complex to control the development of multicellular tomato trichomes, which differs from a set of gene regulation including GIS–MYB–bHLH–WD40 in Arabidopsis; however, the role of this Hair–Wo–SlCycB2 complex in regulating multicellular trichome formation might be somewhat conserved in the *Solanaceae* (Chang et al., 2018). Hair2 (H2), the closest homolog of Hair, is also involved in trichome development in tomato. Compared to the wild type, in the knockout *h2* mutant, both the number and length of type I trichomes are significantly reduced in leaves and stems, while the overexpression lines showed the opposite phenotype. Yeast two-hybrid and pull-down assays showed that H2 also interacts with Wo. Luciferase complementation imaging assays confirmed that these proteins directly interact, indicating that H2 and Wo jointly regulate trichome development. These findings indicate that H2 plays an important role in the initiation and elongation of type I trichomes in tomato (Chun et al., 2021).

**Figure 1:**
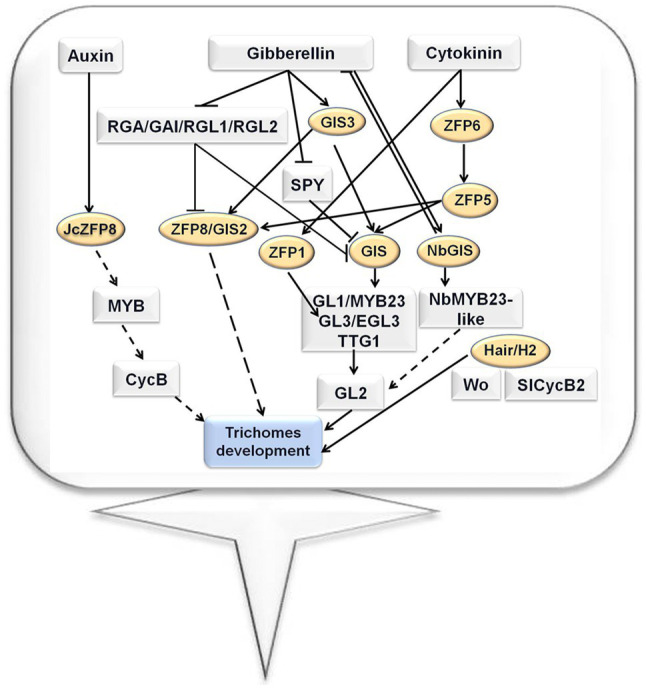
Trichome development regulatory network involving the C2H2 zinc finger proteins in Arabidopsis, tomato, and tobacco (Yan et al., 2014; Sun et al., 2015; Chang et al., 2018; Shi et al., 2018a; Zhang et al., 2020; Chun et al., 2021).

As described above, C2H2 zinc finger proteins play crucial roles in the fate determination, branching, and elongation of trichomes. Plant hormones such as auxin, cytokinin, and gibberellin are involved in this process. For unicellular trichomes in Arabidopsis, GIS3 and ZFP6 regulate a series of downstream C2H2 zinc finger genes, such as *GIS*, *ZFP5*, *ZFP8*, and *GIS2*. These downstream C2H2 zinc finger proteins further regulate the GL1/MYB23-GL3/EGL3-TTG1 complex, thereby determining the fate of trichome. GIS is also involved in regulating the branching of trichomes. For multicellular trichomes of tobacco and tomato, tobacco NbGIS shares a similar role with GIS of Arabidopsis in regulating trichome development. However, in tomato, Hair and H2 play different regulatory roles from C2H2 zinc finger proteins in Arabidopsis: Hair and H2 determine the fate of trichomes by regulating HD-ZIP type transcription factors and cyclin. Finally, H2 is also involved in regulating the elongation process of trichomes. The regulatory networks for Arabidopsis, tobacco, and tomato trichome development involving C2H2 zinc finger proteins are shown in Figure 1.

### Roles of C2H2 Zinc Finger Proteins in Regulating Root Hair Development

Root hairs are specialized structures produced by root epidermal cells (Han et al., 2020b; Zeb et al., 2020). Root hairs expand the contact area between the root and the soil, promote the absorption of water and nutrients, and interact with microorganisms in the environment (Gilroy and Jones, 2000; Huang et al., 2020; Ye et al., 2021).

Root hair development is roughly divided into four stages: the determination of root hair cell fate, root hair initiation, elongation (tip growth), and maturity (Echo and Richard Dong Wook, 2013). Root epidermal cells form root hairs in different manners, depending on the plant species; these models are grouped into three types based on the location of root hair formation. In plants that employ the first model, such as rice (*Oryza sativa*), all root epidermal cells can randomly differentiate into root hair cells. Differentiated root hair cells separate from non-root hair cells, forming a unique spacing pattern (Glover, 2000). In plants that employ the second model, such as *Brachypodium* grasses, asymmetric epidermal cells form during the last cell division before leaving the meristem. The small daughter cells differentiate to form root hair cells, and the large daughter cells form pavement cells: The two types of cells alternate along the longitudinal root axis (Marzec et al., 2014). In plants that employ the third model, such as Arabidopsis, the identity of root hair cells is determined by the cell’s location; for example, epidermal cells in contact with only one cortical cell (“N” position) will differentiate into pavement cells, while epidermal cells in contact with two cortical cells (“H” position) will differentiate into root hair cells (Salazar-Henao et al., 2016).

The molecular mechanisms underlying the third model of root hair development have been fully elucidated in Arabidopsis. A series of proteins, such as bHLH transcription factors, MYB-type transcription factors, WD40 repeat proteins, HD-ZIP transcription factors, and C2H2 zinc finger proteins function as key regulators during root hair fate determination (Shibata and Sugimoto, 2019; Vissenberg et al., 2020). The downstream C2H2 zinc finger proteins and bHLH transcription factors play important roles in the initiation and elongation of root hairs (Salazar-Henao et al., 2016; Feng et al., 2017; Han et al., 2020b).

The C2H2 zinc finger protein ZFP5 not only plays an important role in trichome development, but it is also a key regulator of root hair initiation and morphogenesis. Gene expression analysis indicated that *ZFP5* is widely expressed in roots, stems, branches, and young leaves; moreover, *in situ* hybridization revealed that *ZFP5* is mainly expressed in roots and preferentially expressed in root hair cells. ZFP5 is an active regulator of Arabidopsis root hair development; *zfp5* mutants and *ZFP5*:RNAi lines produced fewer and shorter root hairs than the wild type. Genetic and molecular experiments revealed that ZFP5 affects root hair development by directly promoting the expression of *CPC* (An et al., 2012). In addition, the expression of *ZFP5* is induced by cytokinin and ethylene, allowing ZFP5 to mediate root hair development in response to these hormones (An et al., 2012). ZFP5 also regulates root hair development *via* the GA signaling pathway; for example, in the absence of phosphorus and potassium, the GA signaling pathway induces *ZFP5* expression and promotes root hair elongation (Huang et al., 2020).

In addition to these positive regulators, AtZP1 is a negative regulator of root hair development, inhibiting the initiation and elongation of root hairs. *AtZP1* is strongly expressed in root hair cells, and AtZP1 has transcriptional inhibitory activity. Arabidopsis plants overexpressing *AtZP1* lacked root hairs, while the *atzp1* loss-of-function mutants had longer and more numerous root hairs than the wild type. AtZP1 directly binds to the promoter region of the root hair initiation gene *RHD6*, as well as the root hair extension genes *ROOT HAIR DEFECTIVE6-LIKE2* (*RSL2*) and *RSL4*, to negatively regulate root hair growth. *AtZP1* is regulated by the upstream transcription factor GL2, whose activation or inhibition determines the fate of root hair development. AtZP1 thus negatively regulates the initiation and elongation of root hairs *via* the transcriptional regulation of the GL2/ZP1/RSL (RHD6, RSL4, and RSL2) signaling pathway (Han et al., 2020b).

**Figure 2:**
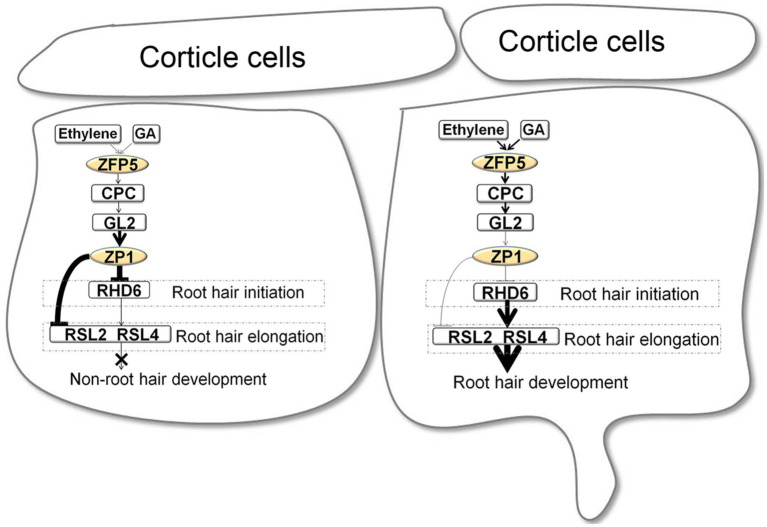
Root hair development regulatory network involving the C2H2 zinc finger proteins in Arabidopsis (An et al., 2012; Han et al., 2020b).

The above studies indicate that C2H2 zinc finger proteins play pivotal roles in the fate determination, initiation, and elongation of Arabidopsis root hairs. During root hair development, ZFP5 determines root hair fate by directly binding to the promoter of *CPC via* the ethylene and GA signaling pathways. The downstream protein AtZP1 controls the initiation and elongation of root hairs by directly binding to the promoters of *RHD6*, *RSL4*, and *RSL2*. The Arabidopsis root hair development regulatory network involving C2H2 zinc finger proteins is shown in Figure 2.

### Roles of C2H2 Zinc Finger Proteins in Regulating Salt Gland Development

Halophytes can complete their lifecycles in extremely saline soils containing NaCl levels of 200mM or more (Meng et al., 2018). Some halophytes, i.e., recretohalophytes, have evolved salt glands, which expel salt out of the plant body to maintain the proper salt balance (Feng et al., 2014; Leng et al., 2019a; Yuan et al., 2019a). Salt glands are mainly distributed on the surfaces of the stems and leaves of recretohalophytes (Leng et al., 2018, 2019b).

Salt glands are divided into two types based on their structures: two-celled salt glands and multi-celled salt glands (Yuan et al., 2019a). Two-celled salt glands are generally found in monocotyledonous gramineous plants such as *Spartina anglica* and have a relatively simple structure; each gland generally comprises only two cells, a salt-collecting basal cell and a salt-secreting cap cell (Yuan et al., 2016a). Multicellular salt glands, which are usually found in dicotyledonous recretohalophytes such as *L. bicolor*, have a more complicated structure (Yuan et al., 2016b). The salt gland complex consists of several salt-collecting cells (stalk cells or basal cells) and several salt-secreting cells. The number of cells that make up the salt gland complex varies depending on the species (Dassanayake and Larkin, 2017).

Many studies of the development and functions of salt glands have focused on the multicellular glands of *Limonium*, starting with preliminary research on the development of salt glands in *Limonium latifolium* (Arisz et al., 1955) and *Limonium vulgare* (Faraday and Thomson, 1986). Wiehe and Breckle first proposed a developmental model for *Limonium* salt glands describing how these glands begin as a single proto-epidermal cell that undergoes five divisions to generate a 20-celled salt gland complex (Wiehe and Breckle, 2015). Recent studies have increasingly focused on salt gland development and secretion in *L. bicolor*, which produces multicellular salt glands on its leaves and stems but lacks trichomes (Li et al., 2020). By contrast, non-halophytes such as Arabidopsis, tomato, and tobacco produce trichomes but no salt glands.

Arabidopsis trichomes have been systematically studied as a model of epidermal cell differentiation (Proust et al., 2016). These trichomes are arranged at specific intervals of the smallest distance, also known as the spacing pattern. Interestingly, the distribution pattern of the salt glands in *L. bicolor* is quite similar to that of Arabidopsis, as they are also arranged in an interval pattern (Leng et al., 2018). In addition, like trichomes in Arabidopsis, salt glands are the earliest epidermal structures to be produced on *Limonium* leaves (Yuan et al., 2015). Therefore, salt glands and trichomes are thought to have evolved from the same ancestral structure in different species. The key genes involved in the development of these epidermal structures may therefore be homologous. To test this hypothesis, RNA sequencing was performed on *L. bicolor* leaves at different stages of salt gland development, and the resulting data were compared with the Arabidopsis genome database (Yuan et al., 2015). Numerous *L. bicolor* genes that were differentially expressed at different stages of salt gland development were found to be homologous to the key genes involved in trichome development in Arabidopsis, including C2H2 zinc finger genes, laying the foundation for the further mining of key genes involved in salt gland development.

To identify C2H2 genes involved in the early stages of salt gland development, we searched for C2H2 zinc finger genes in *L. bicolor* with homologs in Arabidopsis that function in trichome development with high levels of expression specifically during this process. We identified and cloned the genes *LbGIS*, *LbGIS2*, *LbZFP8*, *LbZFP5*, *LbGIS3*, and *LbZFP6*. These C2H2 zinc finger genes may play important roles in salt gland development. Tobacco and tomato trichomes are multicellular, which is similar to the multicellular salt glands of Limonium. Therefore, we analyzed and compared the amino acid sequences of the C2H2 zinc finger proteins of *L. bicolor* with the C2H2 zinc finger proteins that regulate trichome development in Arabidopsis, tobacco, and tomato; these amino acid sequences are shown in Supplementary Table 1.

**Figure 3:**
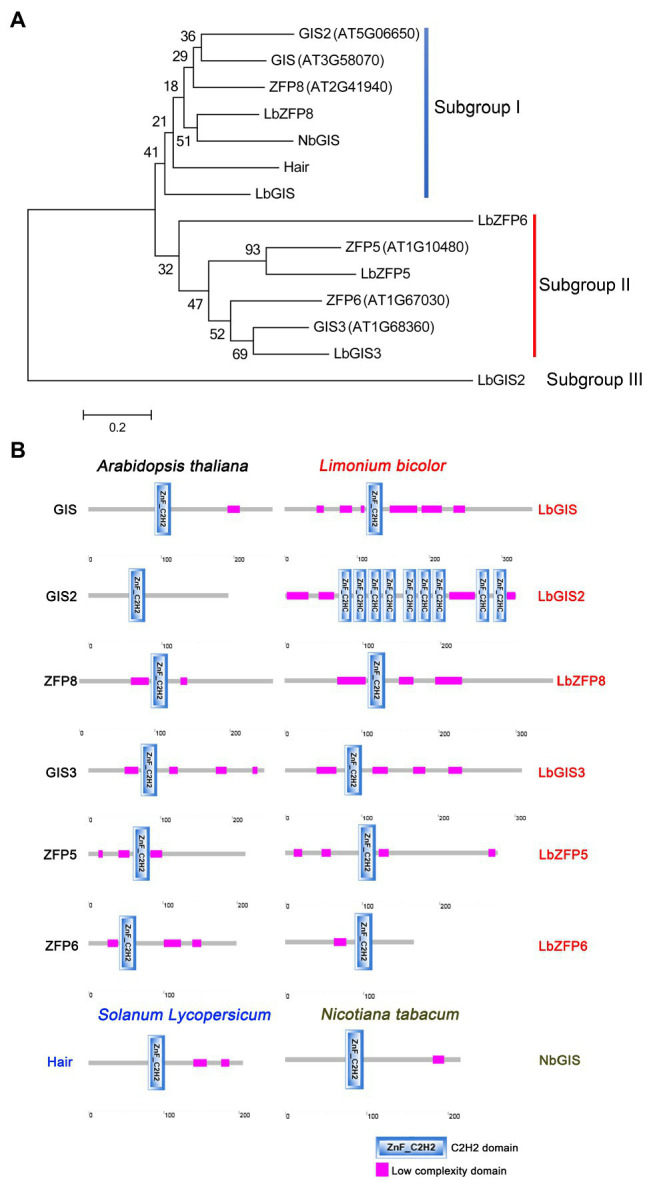
Phylogenetic tree **(A)** and protein domain analysis **(B)** of the C2H2 homologous proteins related to trichomes and salt gland development in Arabidopsis, tabacco, tomato, and Limonium.

Phylogenetic analysis showed that GIS2, GIS, ZFP8, LbZFP8, NbGIS, Hair, and LbGIS clustered together as subgroup I, with LbGIS and NbGIS being the most closely related proteins of this subgroup (Figure 3A). In subgroup II, LbZFP6, ZFP5, LbZFP5, ZFP6, GIS3, and LbGIS3 clustered together, with LbGIS3 and AtGIS3, ZFP5, and LbZFP5 being the most closely related proteins of this subgroup (Figure 3A). LbGIS2 is located in a separate clade (subgroup III) from the other proteins analyzed, which may be related to the specificity of its protein structure and function (Figure 3A). These results indicate that the C2H2 zinc finger proteins in *L. bicolor* share a close evolutionary relationship with the zinc finger proteins that control trichome development in non-halophytes. In particular, in subgroup I, LbZFP8 in *L. bicolor* is closely related to NbGIS in tobacco. NbGIS controls the differentiation of multicellular secretory trichomes in tobacco, suggesting that LbZFP8 likely controls the development of multicellular salt glands in *L. bicolor* (Figure 3A).

Amino acid sequence analysis indicated that most homologous proteins in *Limonium* have relatively long sequences (except for LbZFP6) and that all homologous C2H2 zinc finger proteins in Limonium, Arabidopsis, tobacco, and tomato contain typical C2H2 zinc finger domains. Further analysis revealed that Limonium proteins contain more low-complexity domains than the others (Supplementary Table 2), which may be related to the development of *L. bicolor* salt glands. *LbGIS2* encodes a protein with nine C2H2 domains and no QALGGH sequences, which is significantly different from the other proteins in this family (Figure 3B; Supplementary Table 2); this explains why this protein forms a different subgroup in the phylogenetic tree. The differences between the homologous protein sequences of Arabidopsis, tobacco, and tomato likely explain why *Limonium* develops salt glands while the remaining species form trichomes; this hypothesis is currently being investigated in our laboratory.

## Outlook and Conclusion

C2H2 zinc finger proteins are integral components of the regulatory networks controlling plant epidermal cell development. These proteins interact with other factors and play unique roles in controlling trichome, root hair, salt gland, and stomatal development. The roles of C2H2 zinc finger proteins in trichome and root hair development have been extensively studied: The tissue-specific expression patterns of these proteins in Arabidopsis (GIS, GIS2, GIS3, ZFP1, ZFP5, ZFP6, ZFP8, and AtZP1), their roles in epidermal cell development, and associated references are shown in Supplementary Table 3. However, the roles of these proteins in the development of stomata and salt glands have not been reported. Stomatal development is relatively well characterized, but the pathways involving C2H2 zinc finger proteins require further study. Specifically, the genes encoding C2H2 zinc finger proteins involved in stomatal development must be identified and their functions further examined. *Limonium bicolor* salt glands are multicellular, which is quite different from the unicellular structure of trichomes in Arabidopsis and the other multicellular trichomes of tobacco and tomato. The development of salt glands appears to be homologous to trichome development, suggesting that homologous genes might control these two processes. C2H2 zinc finger proteins are known to regulate the development of multicellular trichomes and may therefore also control the development of multicellular salt glands, but their specific roles and the underlying molecular mechanisms require further study. The possibility that unique genes control salt gland development requires further investigation.

Although different plants and different types of C2H2 zinc finger proteins have varied responses to hormones and developmental signals, some proteins have similar functions and appear to be interchangeable in the initial control of epidermal structure; for example, ZFP6 in the GIS family regulates trichome initiation by integrating GA and cytokinin signaling in a manner similar to that of ZFP5 (Zhou et al., 2013). Homologous genes in different species could also have similar functions, as demonstrated by the heterologous expression of genes involved in the development of epidermal structures in other species (Shi et al., 2018b); however, some genes function in specific signaling pathways (Chang et al., 2018). The development of salt glands may also require C2H2 zinc finger proteins homologous to those that regulate the development of other epidermal structures, inspiring us to study the development of salt glands in the future.

To date, only C2H2 zinc finger proteins that play positive roles in trichome development have been reported, and no negative regulators have been identified; thus, the possibility that a C2H2 transcription factor negatively regulates trichome development requires further exploration. With the rapid rise of new experimental technologies, such as single cell sequencing and systems biology, we will no doubt gain further insights into the roles of the C2H2 zinc finger protein family in epidermal cell differentiation in plants.

## Author Contributions

GH wrote this manuscript. YL, ZQ, CW, YZ, JG, and MC participated in the writing and modification of this manuscript. GH and BW conceptualized the idea. All authors contributed to the article and approved the submitted version.

## Funding

This work was supported by National Natural Science Research Foundation of China (project nos. 32000209, 31770288, and 31800304), Natural Science Research Foundation of Shandong Province (project nos. ZR2020QC031 and ZR2019MC065), and China Postdoctoral Science Foundation (project no. 2020M672114).

## Conflict of Interest

The authors declare that the research was conducted in the absence of any commercial or financial relationships that could be construed as a potential conflict of interest.

## Publisher’s Note

All claims expressed in this article are solely those of the authors and do not necessarily represent those of their affiliated organizations, or those of the publisher, the editors and the reviewers. Any product that may be evaluated in this article, or claim that may be made by its manufacturer, is not guaranteed or endorsed by the publisher.

## Footnotes

^1^ https://www.arabidopsis.org/

^2^ https://www.ncbi.nlm.nih.gov/

^3^ http://smart.embl-heidelberg.de/
