# Supplementary material for: Advances in the Regulation of Epidermal Cell Development by C2H2 Zinc Finger Proteins in Plants
Source: Front Plant Sci. 2021 Sep 24;12:754512. doi: 10.3389/fpls.2021.754512 (PMC8497795; doi:10.3389/fpls.2021.754512)
Supplement: Supplementary file 1 [file Table_1.docx]

**Supplementary Table 1** Amino acid sequence of C2H2 zinc finger proteins involved in the development of epidermal cells in *Arabidopsis thaliana*, *Limonium bicolor*, *Solanum Lycopersicum* and *Nicotiana tabacum*.

| Proteins | Amino acid sequence |
| --- | --- |
| **GIS** | MDEATGETETQDFMNVESFSQLPFIRRPKDKNPKPIRVFGKDFTGRDFSITTGQEDYTDPYQTKNKEEEE EEDQTGDNST DNNSISHNRR FECHYCFRNFPTSQALGGHQ NAHKRERQLA KRGVSSYFYH PDNNPYSYRH YPSWTNGPLTAARSYGGFSS GPKPSGYYTR PSYGSQLGLW RLPPRVQGVY NSNAAFTSNGSSSSSNSTLP LLTRSQTQLS SQVGGSAAQN RMSSYGYGLS PNVQDHVSLD LHL |
| **GIS2** | MKTYDFMNVN SFSPKERPIR LFGFEFGASH EESESKDNYN ENNESIKDDN KEKRFKCHYC FRNFPTSQAL GGHQNAHKRE RQQTKRFNLH SNAAAFFHRQ QNHIAASRLY EDRYSLEAVQ INDARLGLCR MYNSSASFNR DRSSYYNRYI PWFIGDHQTR PTYVGGGSSS HGLFYESKKN VPDHVSLDLR L |
| **ZFP8** | MDETNGRRET HDFMNVNVES FSQLPFIRRT PPKEKAAIIR LFGQELVGDN SDNLSAEPSD HQTTTKNDES SENIKDKDKE KDKDKDKDNN NNRRFECHYC FRNFPTSQAL GGHQNAHKRE RQHAKRGSMT SYLHHHQPHD PHHIYGFLNN HHHRHYPSWT TEARSYYGGG GHQTPSYYSR NTLAPPSSNP PTINGSPLGL WRVPPSTSTN TIQGVYSSSP ASAFRSHEQE TNKEPNNWPY RLMKPNVQDH VSLDLHL |
| **ZFP5** | MSINPTMSRT GESSSGSSSD KTIKLFGFEL ISGSRTPEIT TAESVSSSTN TTSLTVMKRH ECQYCGKEFA NSQALGGHQN AHKKERLKKK RLQLQARRAS IGYYLTNHQQ PITTSFQRQY KTPSYCAFSS MHVNNDQMGV YNEDWSSRSS QINFGNNDTC QDLNEQSGEM GKLYGVRPNM IQFQRDLSSR SDQMRSINSL DLHLGFAGDA A |
| **GIS3** | MEELDFSSKT TTSRLKLFGF SVDGEEDFSD QSVKTNLSSV SPERGEFPAG SSGRSGGGVR SRGGGGGGGE RKYECQYCCR EFGNSQALGG HQNAHKKERQ QLKRAQLQAT RNAAANFSNA GSASQFLRNP IVSAFAPPPH LLSSSAVPQP MGGPWMYLPR VSPSQLHVSH GCVIQDGSGG AGAGGFSYEY GARDSGFGVV GAQMRHVQAH GPRPSVNGFS REVGTTFDDG LGLDLHLSLA PAGH |
| **ZFP6** | MATETSSLKL FGINLLETTS VQNQSSEPRP GSGSGSESRK YECQYCCREF ANSQALGGHQ NAHKKERQLL KRAQMLATRG LPRHHNFHPH TNPLLSAFAP LPHLLSQPHP PPHMMLSPSS SSSKWLYGEH MSSQNAVGYF HGGRGLYGGG MESMAGEVKT HGGSLPEMRR FAGDSDRSSG IKLENGIGLD LHLSLGP |
| **LbGIS** | MDRRDIETHDFMNVESFSQLPFIRPAPLKEKTAIRIFCKELGGRDASKSVDANNHSDDQESKDST VNVSDNRKFVCNYCCRNFPTPQALGGHQNAHKRERQNAKRAQRQTPKFHIGGTYLRSSWSSTTR FYGSRHVYNGSHYSQAHTINGSALAFWRSHSSPLSNYARDPSKMIDPLPVYVNGNL NINNSSISVSRFGYEQKQEVQDHVSLDLHL |
| **LbGIS2** | MSSRSRSRSRSRSRSRSRSRSRSPRDRRMRTERTYSYREAPYRRDGRDGR GDGRDGRDGRRGGLSQNNLCNNCKKPGHFARECPNVAVCNNCGLPGHIA AECTTKALCWNCREPGHMASNCPNEGICHSCGKAGHRARECPRPEVPHG DVRLCNNCFKPGHLAAECTNDKACKNCRKTGHLARDCQNDPICNLCNVG GHLARQCPKANVIGDRGGGGRYGGFGGGGGFGGGGGFGGGGGGGYRD VVCRSCNQMGHMSRDCMGGSQMVICHNCGGRGHMAFECPSGRMMDR GGGLRRY |
| **LbZFP8** | MEKAETHDFMNVESFSQLPFIRPAPSTNKEKTPIRLFGIEFGNNSAATTTDEGAESNSTTSE DQPKETTTTSENNQNSATNNNNTSYGDTGTGGGGGGTSSSSSGRKFECHYCCRNFPTSQA LGGHQNXHKKERQHAKRAHLQSAMLHSGXHYHHHHLGGTTTADHHYLYGSLLGYHHRLG GGTSTPTISQIPHNSNFLNSSASSSPWSSNYNNTSSFSSSGSASTANYNRYYYPTSSGYNHH HTTAATYSNHHISSPPPINGNPLSTWRNNVNSTPAAVQARHHHHHAGSSRDRLMQQQQQY PCFPSMLRNESPNSSTAQIDVVNGSVLEGRSTKSLQDHVSLDLHL |
| **LbZFP5** | MVKEAYLPLFSLSSEHSSRSSSDQTKRLKLFGIELDLNEHKACKGEDSSSVSPSDQDESMNSS HTGSVESRDDDKEKASSFTTGSMVVVGEEKKFECQFCFKGFANSQALGGHQNAHKKERLKRK RLQLQAXKASFGFCLNQHQQHPYQNHLINYLVDQQQLQLQHGSSTSTVWYYDTTSSPQDAS EFKESQISFCSADQDRNHEHHQPGGFKYIFPAQDQTHQRQFTLTGEANNNQCSRPLAVKPSS VLPIAKQSCKSVLDLGLGLGLGLGSSV |
| **LbGIS3** | MAELDDDFHHHQPSTNDLKLFGFKVTGTSSYHHHHQQQEDDAVSSSASPSTSPDPPLDHH HHHPATTNPIPSHDGRRFECQYCYREFANSQALGGHQNAHKKERQQLKRAQLQASRNAAAA AAAAASSPSLYASCYLRSNPIISAFAPSPPHLLPSTASGSPPILIPAAMSGSAGSPPSNSSSWAY MPRAAQPLHVSHGCVFPTSPPNGRPPGMMMMMGSGGVATGSGTGMKPVQYAGVGGFSTDS AVAASFNHHHHHHQINGVPRGGGAESVDGSSTSLSRFSMLPNLEHDSVGIDLHLSLGPAGSSL |
| **LbZFP6** | MLSPIHIVAQTFRREIDTYPFARSLVYIDGQYCLPANGSNTGSSADEWLNLSLGGGQLDDTN NYGGHHHGYGNGHVHHAPSSVSPKKFFKCKYCTRKFLNSRALGGHQNAHKKERGEVRRFHM ERSLTMGMPYHHQNKPTTAVVQSIEVEPHALLHGGQTAHEV |
| **Hair** | MEKIGREAVDYMNMKSFSQPLRKKSIRLFGKEFSVGDSTNMSEST DKNPLHHEPKPNTMSISANRIDKTGHVDEISRKYECYYCFRSFPT SQALGGHQNAHKKERQNAKLSHLQSSIVHETNRNRFGEPSTAA TRLTHYHSTWSNINNNNVYSPNYNEAFWQIPPTIHHYQNNINP PSSFSHDSFFPNDEEKRE VQNHVSLDLHL |
| **NbGIS** | MDRRDIETHDFMNVESFSQLPFIRPAPLKEKTAIRIFCKELGGRDASKSVDANNHSDDQESK DSTVNVSDNRKFVCNYCCRNFPTPQALGGHQNAHKRERQNAKRAQRQTPKFHIGGTYLRSS WSSTTRFYGSRHVYNGSHYSQAHTINGSALAFWRSHSSPLSNYARDPSKMIDPLPVYVNGNLN INNSSISVSRFGYEQKQEVQDHVSLDLHL |
