# Supplementary material for: Advances in the Regulation of Epidermal Cell Development by C2H2 Zinc Finger Proteins in Plants
Source: Front Plant Sci. 2021 Sep 24;12:754512. doi: 10.3389/fpls.2021.754512 (PMC8497795; doi:10.3389/fpls.2021.754512)
Supplement: Supplementary file 2 [file Table_2.docx]

**Supplementary Table 2** Protein sequence analysis of C2H2 zinc finger proteins involved in the development of epidermal cells in *Arabidopsis thaliana*, *Limonium bicolor*, *Solanum Lycopersicum* and *Nicotiana tabacum*.

| Proteins | Number of amino acids | Number of C2H2 domains | Number of QALGGH sequences | Number of low complexity domains | [Species](javascript:;) |
| --- | --- | --- | --- | --- | --- |
| GIS | 250 | 1 | 1 | 1 | *Arabidopsis thaliana* |
| GIS2 | 191 | 1 | 1 | 0 | *Arabidopsis thaliana* |
| ZFP8 | 257 | 1 | 1 | 2 | *Arabidopsis thaliana* |
| ZFP5 | 211 | 1 | 1 | 3 | *Arabidopsis thaliana* |
| GIS3 | 244 | 1 | 1 | 4 | *Arabidopsis thaliana* |
| ZFP6 | 197 | 1 | 1 | 3 | *Arabidopsis thaliana* |
| LbGIS | 342 | 1 | 1 | 6 | *Limonium bicolor* |
| LbGIS2 | 297 | 9 | 0 | 4 | *Limonium bicolor* |
| LbZFP8 | 348 | 1 | 1 | 3 | *Limonium bicolor* |
| LbZFP5 | 275 | 1 | 1 | 4 | *Limonium bicolor* |
| LbGIS3 | 309 | 1 | 1 | 4 | *Limonium bicolor* |
| LbZFP6 | 164 | 1 | 0 | 1 | *Limonium bicolor* |
| Hair | 205 | 1 | 1 | 2 | *Solanum Lycopersicum* |
| NbGIS | 215 | 1 | 1 | 1 | *Nicotiana tabacum* |
