# Supplementary material for: Advances in the Regulation of Epidermal Cell Development by C2H2 Zinc Finger Proteins in Plants
Source: Front Plant Sci. 2021 Sep 24;12:754512. doi: 10.3389/fpls.2021.754512 (PMC8497795; doi:10.3389/fpls.2021.754512)
Supplement: Supplementary file 3 [file Table_3.docx]

**Supplementary Table 3** C2H2 zinc finger proteins involved in the development of epidermal cells in *Arabidopsis thaliana*

| Gene name | Expressed tissues | Functions | References |
| --- | --- | --- | --- |
| *GIS* | stem epidermis and flower meristem | trichome fate determination and trichome branching development | ([Gan et al., 2006](#_ENREF_2);[Gan et al., 2007a](#_ENREF_3);[Sun et al., 2013](#_ENREF_7)) |
| *GIS2* | primary and secondary meristems, developing flowers and successive cauline leaves | trichome fate determination | ([Gan et al., 2007b](#_ENREF_4)) |
| *ZFP8* | cauline leaves | trichome fate determination | ([Gan et al., 2007b](#_ENREF_4)) |
| *ZFP5* | roots, stems, branches, and young leaves | trichome fate determination and root hair fate determination | ([An et al., 2012](#_ENREF_1);[Zhou et al., 2012](#_ENREF_9)) |
| *GIS3* | root, the first and second developing branch and fully elongated first internode of the main stem | trichome fate determination | ([Sun et al., 2015](#_ENREF_6)) |
| *ZFP6* | root, the first and second developing branch and fully elongated first internode of the main stem | trichome fate determination | ([Zhou et al., 2013](#_ENREF_10)) |
| *ZFP1* | flower, main stem and silique | trichome fate determination | ([Zhang et al., 2020](#_ENREF_8)) |
| *AtZP1* | root hair, trichome, young leaves, flowers, siliques | root hair initiation and elongation | ([Han et al., 2020](#_ENREF_5)) |

**References**

An, L., Zhou, Z., Sun, L., Yan, A., Xi, W., Yu, N., Cai, W., Chen, X., Yu, H., and Schiefelbein, J. (2012). A zinc finger protein gene ZFP5 integrates phytohormone signaling to control root hair development in Arabidopsis. *The Plant Journal* 72**,** 474-490.

Gan, Y., Kumimoto, R., Liu, C., Ratcliffe, O., Yu, H., and Broun, P. (2006). GLABROUS INFLORESCENCE STEMS modulates the regulation by gibberellins of epidermal differentiation and shoot maturation in Arabidopsis. *Plant Cell* 18**,** 1383-1395.

Gan, Y., Liu, C., Yu, H., and Broun, P. (2007a). Integration of cytokinin and gibberellin signalling by Arabidopsis transcription factors GIS, ZFP8 and GIS2 in the regulation of epidermal cell fate. *Development* 134**,** 2073-2081.

Gan, Y., Liu, C., Yu, H., and Broun, P. (2007b). Integration of cytokinin and gibberellin signalling by Arabidopsis transcription factors GIS, ZFP8 and GIS2 in the regulation of epidermal cell fate. *Development* 134**,** 2073-2081.

Han, G., Wei, X., Dong, X., Wang, C., Sui, N., Guo, J., Yuan, F., Gong, Z., Li, X., Zhang, Y., Meng, Z., Chen, Z., Zhao, D., and Wang, B. (2020). Arabidopsis ZINC FINGER PROTEIN1 Acts Downstream of GL2 to Repress Root Hair Initiation and Elongation by Directly Suppressing bHLH Genes. *Plant Cell* 32**,** 206-225.

Sun, L., Zhang, A., Zhou, Z., Zhao, Y., Yan, A., Bao, S., Yu, H., and Gan, Y. (2015). GLABROUS INFLORESCENCE STEMS3 (GIS3) regulates trichome initiation and development in Arabidopsis. *New Phytologist* 206**,** 220-230.

Sun, L.L., Zhou, Z.J., An, L.J., An, Y., Zhao, Y.Q., Meng, X.F., Steele-King, C., and Gan, Y.B. (2013). GLABROUS INFLORESCENCE STEMS regulates trichome branching by genetically interacting with SIM in Arabidopsis. *J Zhejiang Univ Sci B* 14**,** 563-569.

Zhang, A., Liu, Y., Yu, C., Huang, L., Wu, M., Wu, J., and Gan, Y. (2020). Zinc Finger Protein 1 (ZFP1) Is Involved in Trichome Initiation in Arabidopsis thaliana. *Agriculture* 10**,** 645.

Zhou, Z., An, L., Sun, L., and Gan, Y. (2012). ZFP5 encodes a functionally equivalent GIS protein to control trichome initiation. *Plant Signal Behav* 7**,** 28-30.

Zhou, Z., Sun, L., Zhao, Y., An, L., Yan, A., Meng, X., and Gan, Y. (2013). Zinc Finger Protein 6 (ZFP6) regulates trichome initiation by integrating gibberellin and cytokinin signaling in Arabidopsis thaliana. *New Phytol* 198**,** 699-708.
